# Supplementary material for: Endophytes from Halotolerant Plants Aimed to Overcome Salinity and Draught
Source: Plants (Basel). 2022 Nov 6;11(21):2992. doi: 10.3390/plants11212992 (PMC9658857; doi:10.3390/plants11212992)
Supplement: Supplementary file 1 [file plants-11-02992-s001.zip › plants-1941549-supplementary.pdf]

Supplementary Materials

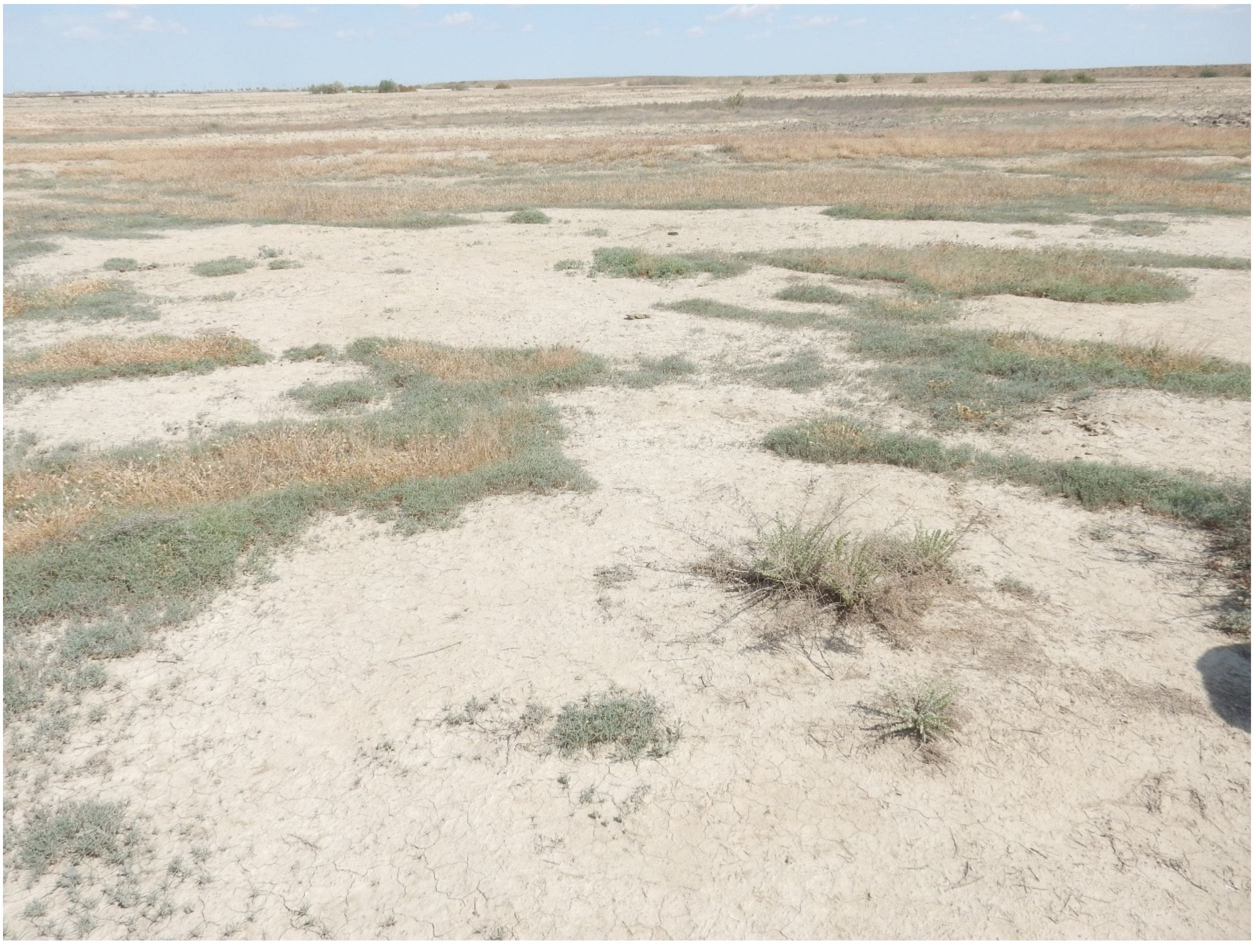

Figure S1. Place near the village of Winter Rate Neftekumsky district, Stavropol Territory, 44.5453 N 45.1907 E.

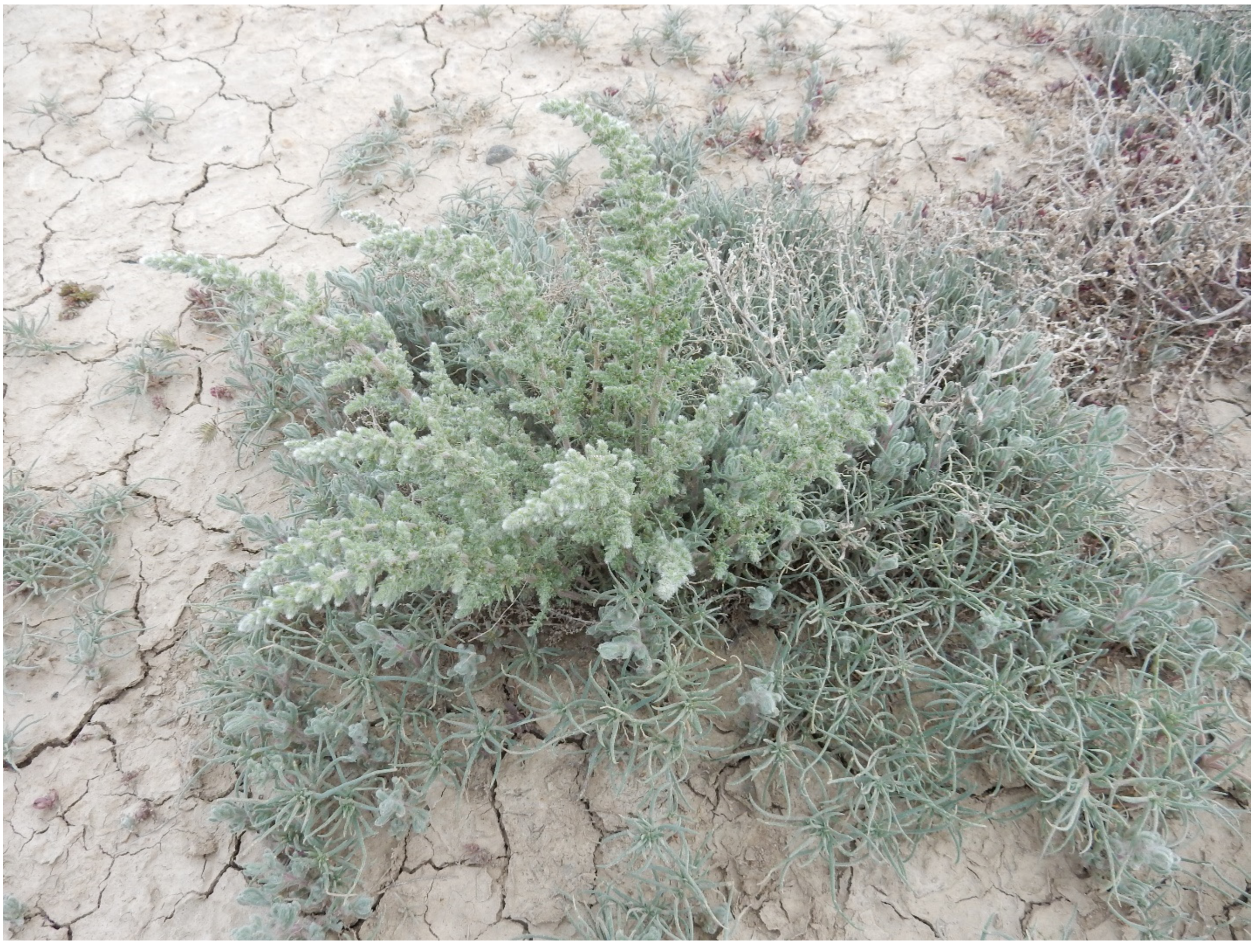

Figure S2. Plants *Salicornia europaea* L., *Salsola australis* R.Br. and *Bassia sedoides* (Pall.) Asch., growing in one place.

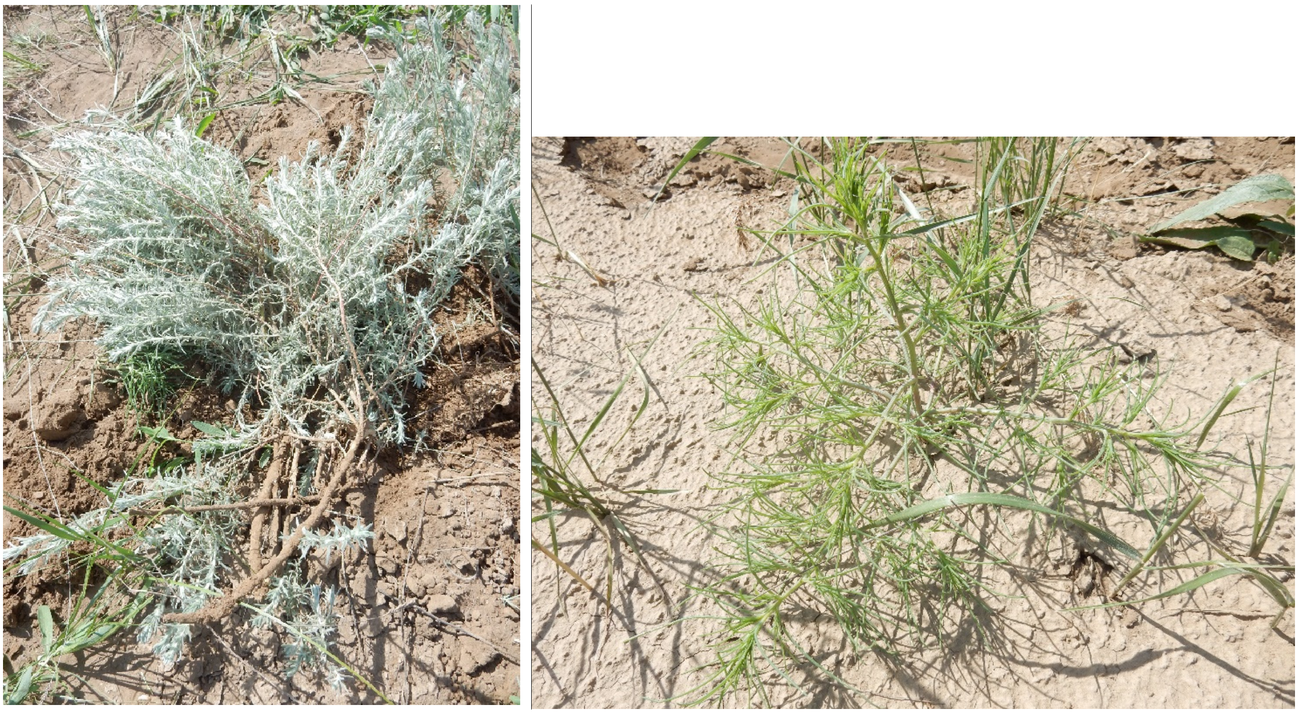

Figure S3. Place near the village of Burgun-Majary (Kurgan) Levokumsky district, Stavropol Territory, 44.5017 N 44.2901 E. *Kochia prostrata* (L.) Schrad A (left) and *Salsola australis* R.Br. (right).
